# Susceptibility loci for pancreatic cancer in the Brazilian population

**DOI:** 10.1186/s12920-021-00956-5

**Published:** 2021-04-20

**Authors:** Mateus Nóbrega Aoki, Angelika Stein, Jaqueline Carvalho de Oliveira, Roger Chammas, Miyuki Uno, Francielle Boçon de Araújo Munhoz, Anelis Maria Marin, Federico Canzian

**Affiliations:** 1 Laboratory for Applied Science and Technology in Health, Carlos Chagas Institute, Oswaldo Cruz Foundation (Fiocruz), Curitiba, PR Brazil; 2 Genomic Epidemiology Group, German Cancer Research Center (DKFZ), Heidelberg, Germany; 3 Department of Genetics, Federal University of Parana, Curitiba, Brazil; 4 Departamento de Radiologia E Oncologia, Centro de Investigação Translacional Em Oncologia, Instituto Do Câncer Do Estado de São Paulo (ICESP), Faculdade de Medicina da Universidade de São Paulo (FMUSP), São Paulo, Brasil

**Keywords:** Pancreatic cancer, SNP, Brazil, Association study, Genetic susceptibility

## Abstract

**Background:**

Pancreatic adenocarcinoma (PA) is a very aggressive cancer and has one of the poorest prognoses. Usually, the diagnosis is late and resistant to conventional treatment. Environmental and genetic factors contribute to the etiology, such as tobacco and alcohol consumption, chronic pancreatitis, diabetes and obesity. Somatic mutation in pancreatic cancer cells are known and SNP profile by GWAS could access novel genetic risk factors for this disease in different population context. Here we describe a SNP panel for Brazilian pancreatic cancer, together with clinical and epidemiological data.

**Methods:**

78 pancreatic adenocarcinoma and 256 non-pancreatic cancer subjects had 25 SNPs genotyped by real-time PCR. Unconditional logistic regression methods were used to assess the main effects on PA risk, using allelic, co-dominant and dominant inheritance models.

**Results:**

9 SNPs were nominally associated with pancreatic adenocarcinoma risk, with 5 of the minor alleles conferring protective effect while 4 related as risk factor. In epidemiological and clinical data, tobacco smoking, diabetes and pancreatitis history were significantly related to pancreatic adenocarcinoma risk. Polygenic risk scores computed using the SNPs in the study showed strong associations with PA risk.

**Conclusion:**

We could assess for the first time some SNPs related with PA in Brazilian populations, a result that could be used for genetic screening in risk population such as familial pancreatic cancer, smokers, alcohol users and diabetes patients.

## Background

Pancreatic adenocarcinoma (PA), although relative rare, is the seventh leading cause of cancer death worldwide [1] and is one of the cancers with the poorest prognosis, with a five year survival rate close to 10% [2]. A major cause for the poor prognosis is the late diagnosis and the resistance to conventional treatment [3] and, differently from other tumor types, mortality rates for PA are not improving [4, 5]. In Brazil, PA was responsible for more than 11,000 deaths in 2018, and PA incidence and lethality are increasing in the country [6, 7].

Environmental and genetic factors contribute to the etiology of PA, as the consumption of tobacco is an important risk factor [8, 9]. Other risk factors include the excessive consumption of alcohol, chronic pancreatitis, diabetes, obesity and dietary-endocrine factors [10, 11].

Somatic mutation in pancreatic cells is an essential carcinogenesis event and strongly related with genes such as *KRAS*, *CDKN2A*, *TP53*, *SMAD4*. *KRAS* missense mutations include mainly three hot spots: glycine-12 (G12), glycine-12 (G13), or glutamine-61 (Q61), and occurs virtually in all PA cases [7–9]. Individuals with family history of pancreatic cancer have a higher risk of developing the disease and genetic susceptibility may be related to germinal mutations in known genes for hereditary cancer including *CDKN2A, BRCA2, PALB2, STK11* and *PRSS1*[6]. *CDKN2A* mutation are important in sporadic and familial events and it is estimated that this gene is altered in more than 90% of PA, with 0.6–3.3% of cases described to carry deleterious germline mutations in this gene [12, 13]. *BRCA2* also represents a hot-spot for rare variants/mutation for risk factor in PA[14, 15].

Also single nucleotide polymorphisms (SNPs) have been extensively studied for a possible association with the risk of PA, for example, polymorphisms in the cytochrome P450 enzyme (*CYP2A6*) have been linked to an increased risk of sporadic PA (independent of smoking) [16].

More recently, genome-wide association studies (GWAS) have identified common variants associated with risk of PA mainly in North American, European and Asian populations [17–20]. These studies highlight different loci but their frequency and PA risk association in the Brazilian population is unknown.

Based on this, the present study evaluated 25 SNPs, previously associated with PA risk in GWAS to investigate the influence of these loci in the Brazilian population, including 78 patients with pancreatic adenocarcinoma and 256 controls without cancer history. From the analyzed loci, 10 variants were associated with PA risk in some of the models analyzed, highlighting the importance of these regions.

## Methods

### Study population

In this prospective and consecutive study, we used 78 PA patients recruited from 2018 to 2019 with confirmation by histopathology and/or surgery provided from Academic Biobank of Research on Cancer from the University of São Paulo, located in Centro de Investigação Translacional em Oncologia, Instituto do Câncer do Estado de São Paulo (ICESP), São Paulo, Brazil. The Biobank protocol was approved by the Local Ethics Committee (CEP no. 031/12 and National Ethics Committee (CONEP no.023/2014). As control we used 256 subjects with non-pancreatic cancer, healthy blood donors or orthopedic patients provided from Hospital do Trabalhador, Curitiba PR, Brazil, with Local Ethics Committee (CEP CAAE no. 77979417.8.0000.5248 and 77979417.8.3001.5225) and National Ethics Committee (CONEP 77979417.8.0000.5248) approval. All approvals contemplated demographic and epidemiological data collection for both groups, while for PA cases clinical data were also collected. For all participants, the project was described and informed consent form was obtained in writing format. All the participants had 4 mL of peripheral blood collected and buffy-coat DNA was extracted with QIAmp DNA Blood Mini Kit (QIAGEN) as indicated. A quantification and purity of DNA were performed using NanoDrop One/OneC Microvolume UV Spectrophotometer® (Thermo Scientific).

### SNP selection

For this study we initially selected 26 SNPs reported to be statistically associated with PA susceptibility or survival in previously GWAS studies [18, 21–26] to look for in the Brazilian population: rs11655237, rs2736098, rs351365, rs3790844, rs1486134, rs16986825, rs17688601, rs9581943, rs35226131, rs1561927, rs9854771, rs73328514, rs7310409, rs1517037, rs2853677, rs2941471, rs6971499, rs10991043, rs401681, rs13303010, rs9543325, rs4795218, rs7190458, rs10094872, rs684559 and rs353630. The last two SNPs were selected from a GWAS aimed at finding loci associated with survival of PA patients [26].

### Genotyping

The SNP genotyping was conducted in the Genomic Epidemiology laboratory at the German Cancer Research Center (DKFZ), Heidelberg using TaqMan (ABI, Applied Biosystems, Foster City, CA) and KASP (KBioscence, Hoddesdon, UK) Technologies and TaqMan Genotyping Master Mix (Applied Bioscience) technology, according to the manufacturers’ instructions. All samples were included in a 384-well plate. For quality control, duplicates of 5% of the samples were included. Polymerase chain reaction plates were read on a ViiA7 real time instrument (Applied Biosystems). The ViiA7 RUO Software, version 1.2.2 (Applied Biosystems), was used to determine genotypes. The genotyping concordance between duplicate samples exceeded 99%, and samples with a call rate lower than 75% were discarded from the statistical analysis. rs35226131 was monomorphic in our population, therefore it was not included in further analyses.

### Statistical analysis

Chi-square tests were used to compare sex, ethnicity, smoking and alcohol use, diabetes, pancreatitis between cases and controls, while for age we used t-student test, all conducted with Prism GraphPad. Hardy–Weinberg equilibrium was assessed in control subjects for each polymorphism. For each SNP, the more common allele in controls was assigned as the reference category. All analyses were adjusted for age and sex. Unconditional logistic regression methods were used to assess the main effects for the 25 selected genetic polymorphisms on PA risk, using allelic, co-dominant and dominant inheritance models. We used a *p* < 0.05 threshold to assess statistically significant associations between SNPs and PA risk. Chi-square and Fisher's exact test was used to compare allele frequency between ethnic ancestry from PA patients, controls and reported in database, with statistically significant by *p* < 0.05.

### Polygenic risk score

We used the SNPs investigated in this study to assemble a polygenic risk score (PRS). We included all SNPs except rs684559 and rs353630, which were originally not reported to be associated with PA risk but rather with survival. For each SNP the number of alleles associated with higher PA risk were counted and added up for each study subject, resulting in an unweighted PRS. Additionally, we built a weighted PRS by using the ORs of the original GWASs. For each SNP in the weighted PRS a value of 0 was assigned if 0 risk alleles were present, ln(OR) was assigned if 1 risk allele was present, and 2*ln(OR) if 2 risk alleles were present. Then all the values were summed among them for each subject. Only a subset of the study subjects (67 cases and 228 controls) had a 100% SNP call rate. Therefore, in order to be able to compute comparable score values for all study subjects, we also considered “scaled” scores, in which the PRS values for each subject were multiplied by the ratio between the total number of SNPs and the number of effectively genotyped SNPs for the subject in question. For both PRSs (weighted and unweighted) we calculated quintiles based on the distribution of values in the controls.

The formulas for the unweighted and weighted scores are respectively $\mathop \sum \limits_{1}^{m} aj$ and $\mathop \sum \limits_{1}^{m} aXj$, where *a* = number of risk alleles (0,1,2), m = total number of SNPs, *j* = jth subject, *X* = ln(OR).

Additionally, we created also PRSs using only the 9 SNPs that show association with PA in this population. We analyzed the association between the quintiles of PRSs and PA risk by logistic regression, adjusting for age and sex.

## Results

### Study population data

Table 1 summarizes the epidemiological data for both groups. For the age, PA patients shows a mean age of 62.46 years old and the median age was 62, while for the control group the ages were 56.62 and 57, respectively. Age was not statistically different between cases and controls. The gender distribution was very similar with slightly more females among both cases and controls, which was also not statistically different. About ethnicity, collected as a self-reported variable, European ancestry people were more frequent in both groups (66.7% in PA and 81.3% in controls), while African ancestry people were more frequent in PA than controls (32% and 17.5%, respectively), again not statistically different. Other epidemiological data such as tobacco and alcohol usage, diabetes and personal pancreatitis history are also shown in Table 1. Statistical analysis showed a significant association between tobacco use (*p* = 0.002), diabetes (*p* < 0.0001) and pancreatitis history (*p* < 0.0001) and PA risk while alcohol use and familial PA history was not significant associated.

**Table 1:** Epidemiological data for age, sex, ethnicity distribution, tobacco and alcohol use, diabetes, pancreatitis history and familial history of pancreatic cancer for both groups

	PA cases	Controls	*p* value
Mean age (years)	62.46	56.62	0.0842
Median Age (years)	62	57	–
*Sex*			
Male	41%	43%	0.9560
Female	59%	57%	
*Ethnicity*			
African ancestry	32%	17.5%	0.1303
European ancestry	66.7%	81.3%	
Asian ancestry	1.3%	1.2%	
*Tobacco*			
Yes	51.32	32.31	0.0002
No	48.68	67.69	
*Alcohol*			
Yes	31.58	32.31	0.7774
No	68.42	67.69	
*Diabetes*			
Yes	35.53	9.62	< 0.0001
No	64.47	90.38	
*Pancreatitis history*			
Yes	10.53	0.77	< 0.0001
No	89.47	99.23	

**Fig. 1:**
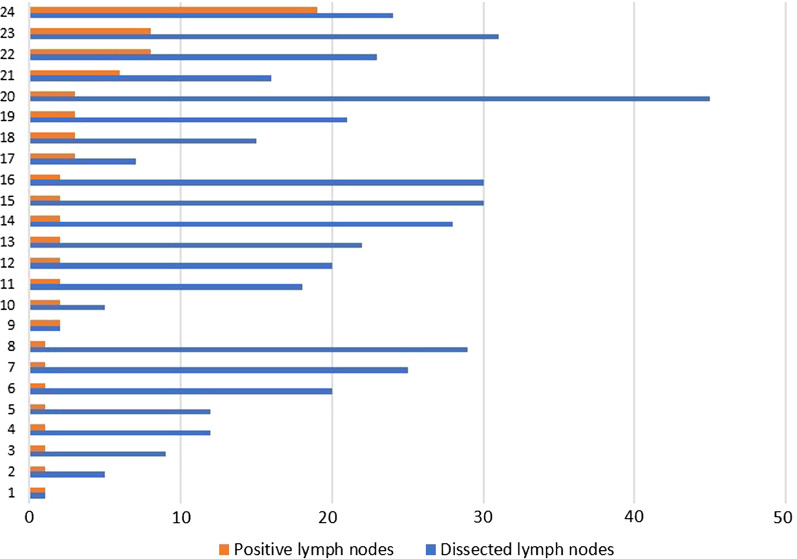
Clinical data for lymph node dissection and positivity in PA patients

When we look for clinical data of the pancreatic cancer patients, almost 75% of then had the tumor located at pancreas head, while 8% was located in pancreatic body and 7% in tail and tail/body. For all 78 pancreatic cancer patients, 38 (49%) were submitted to lymph node dissection, and 24 of then (64%) present positivity with different ratio (Fig. 1). For treatment, 33% of the patients were treated with FOLFIRINOX, while 12% were submitted to surgery and 9% treated with gemcitabine.

For the genotyping analysis, all SNPs were in HWE in controls (*p* > 0.05). Results of association analysis between SNPs and PA risk are shown in Table 2. We found 8 SNPs that were nominally associated with pancreatic cancer risk (*p* < 0.05) with allelic model analysis and one more SNP with both codominant and dominant model analysis. Of these, the minor allele in 5 SNPs showed a protective effect for PA (OR < 1), while for 4 SNPs the minor allele was associated with increase in risk (OR > 1). The most significant findings were related to SNPs rs3790844, rs9854771, rs2941471, rs401681, rs13303010 and rs9543325. For the first one the minor allele represents a protective effect in pancreatic cancer patients. In the same way, in the SNP rs9854771 the minor allele also represents a protective effect. The third SNP where the minor allele represents a protective effect is rs2941471. In a different way, at the SNP rs401681 the minor allele represents a risk factor for pancreatic cancer. At the SNP rs13303010 the minor allele also represents a risk factor for pancreatic cancer, again showed in SNP rs9543325. The complete results for analysis of the SNPs are shown in Table 2.

**Table 2:** SNP associations with PA risk

SNP	Nearest gene	Allele (M/m)^a^	MAF^b^ controls	MAF^b^ cases	Allelic model		Codominant model					Dominant model	
					OR (95% CI)	*p* value	M/M vs M/m		M/M vs m/m			M/M vs M/m + m/m	
							OR (95% CI)	*p* value	OR (95% CI)	*p* value	*p* trend	OR (95% CI)	*p* value
rs11655237	*LINC00673*	C/T	0.13	0.14	1.06 (0.63–1.79)	0.806	1.20 (0.66–2.18)	0.537	0.57 (0.66–4.95)	0.613	0.795	1.14 (0.64–2.05)	0.646
rs2736098	*TERT*	G/A	0.25	0.22	0.79 (0.51–1.22)	0.302	0.68 (0.39–1.20)	0.191	0.85 (0.28–2.54)	0.781	0.361	0.71 (1.41–1.20)	0.208
rs351365	*WNT2B*	G/A	0.29	0.26	0.85 (0.57–1.28)	0.454	0.82 (0.47–1.41)	0.477	0.78 (0.29–2.06)	0.625	0.449	0.81 (0.48–1.36)	0.434
**rs3790844**	*NR5A2*	**T/C**	**0.27**	**0.18**	**0.61 (0.38–0.96)**	**0.036**	**0.56 (0.32–0.99)**	**0.049**	**0.46 (0.12–1.66)**	**0.240**	**0.030**	**0.55 (0.32–0.94)**	**0.031**
rs1486134	*ETAA1*	T/G	0.25	0.24	1.03 (0.68–1.55)	0.867	1.32 (0.45–3.88)	0.610	1.24 (0.43–3.54)	0.42	0.838	1.27 (0.45–3.54)	0.640
rs16986825	*ZNRF3*	C/T	0.17	0.14	0.76 (0.45–1.28)	0.309	0.83 (0.46–1.52)	0.565	0.35 (0.04–2.87)	0.331	0.318	0.78 (0.43–1.40	0.412
**rs17688601**	*SUGCT*	**C/A**	**0.25**	**0.18**	**0.63 (0.40–1.00)**	**0.046**	**1.28 (0.40–4.16)**	**0.685**	**0.63 (0.21–1.88)**	**0.406**	**0.093**	**0.8 (0.27–2.32)**	**0.673**
rs9581943	*PDX1*	G/A	0.36	0.31	1.22 (0.84–1.78)	0.282	0.76 (0.34–1.69)	0.505	1.22 (0.56–2.65)	0.605	0.270	0.98 (0.47–2.05)	0.971
rs1561927	*MIR1208*	T/C	0.24	0.32	1.47 (0.99–2.17)	0.058	1.78 (0.71–4.54)	0.222	2.44 (0.97–6.25)	0.059	0.050	2.12 (1.88–5.26	0.092
**rs9854771**	*TP63*	**G/A**	**0.39**	**0.25**	**0.53 (0.35–0.80)**	**0.003**	**1.09 (0.45–2.70)**	**0.846**	**0.41 (0.17–0.98)**	**0.043**	**0.002**	**0.64 (0.28–1.47)**	**0.288**
rs73328514	*TNS3*	A/T	0.10	0.15	1.56 (0.90–2.70)	0.112	1.35 (0.73–2.49)	0.333	7.94 (0.69–91.22)	0.096	0.098	1.47 (0.81–2.66)	0.203
**rs7310409**	*HNF1A*	**G/A**	**0.41**	**0.49**	**1.41 (0.98–2.00)**	**0.065**	**2.08 (1.08–4.00)**	**0.029**	**2.04 (1.03–4.16)**	**0.043**	**0.061**	**2.08 (1.13–3.84)**	**0.018**
rs1517037	*GRP*	C/T	0.21	0.24	1.18 (0.76–1.82)	0.449	1.35 (0.79–2.32)	0.263	0.94 (0.25–3.52)	0.932	0.434	1.30 (0.77–2.20)	0.313
rs2853677	*TERT*	A/G	0.46	0.37	0.70 (0.48–1.02)	0.068	0.61 (0.34–1.09)	0.100	0.53 (0.24–1.13)	0.104	0.051	0.59 (0.34–1.01)	0.055
**rs2941471**	*HNF4G*	**A/G**	**0.44**	**0.35**	**0.64 (0.43–0.94)**	**0.026**	**0.58 (0.33–1.02)**	**0.063**	**0.44 (0.19–0.99)**	**0.050**	**0.037**	**0.54 (0.32–0.93)**	**0.027**
rs6971499	*LINC-PINT*	A/G	0.11	0.10	1.24 (0.67–2.29)	0.485	0.89 (0.47–1.71)	0.745	–	–	0.523	–	–
rs10991043	*SMC2*	T/C	0.39	0.37	1.10 (0.76–1.60)	0.601	**0.47 (0.22–0.98)**	**0.047**	0.90 (0.43–1.87)	0.789	0.545	0.64 (0.33–1.27)	0.209
**rs401681**	*TERT*	**C/T**	**0.45**	**0.54**	**1.53(1.05–2.22)**	**0.026**	**1.67 (0.86–3.24)**	**0.129**	**2.36 (1.10–5.03)**	**0.026**	**0.026**	**1.86 (0.99–3.50)**	**0.054**
**rs13303010**	*NOC2L*	**A/G**	**0.22**	**0.37**	**1.88 (1.29–2.77)**	**0.001**	**1.39 (0.58–3.33)**	**0.467**	**3.125 (1.29–7.69)**	**0.011**	**0.003**	**1.45 (0.97–5.05)**	**0.061**
**rs9543325**	*13q22.1*	**T/C**	**0.44**	**0.56**	**1.66 (1.15–2.38)**	**0.007**	**2.85 (1.51–5.26)**	**0.001**	**2.63 (1.29–5.26)**	**0.007**	**0.011**	**2.77 (1.56–4.02)**	**0.001**
**rs4795218**	*HNF1B*	**G/A**	**0.20**	**0.11**	**0.53 (0.31–0.90)**	**0.021**	**0.78 (0.15–4.02)**	**0.763**	**0.38 (0.08–1.72)**	**0.209**	**0.015**	**0.44(0.09–2.04)**	**0.295**
rs7190458	*BCAR1*	C/T	0.07	0.08	0.88 (0.42–1.87)	0.755	0.88 (0.42–1.87)	0.755	–	–	0.657	–	–
rs10094872	*MYC*	A/T	0.28	0.34	1.37 (0.92–2.02)	0.111	1.55 (0.90–2.68)	0.112	1.62 (0.65–4.06)	0.295	0.145	1.57 (0.93–2.64)	0.089
rs684559	*CHI3L2*	G/A	0.32	0.29	1.24 (0.84–1.81)	0.270	0.80 (0.34–1.90)	0.627	1.25 (0.55–2.84)	0.586	0.448	1.04 (0.47–2.28)	0.921
rs353630	*CD44*	G/A	0.29	0.30	0.95 (0.64–1.39)	0.806	1.25 (0.49–3.19)	0.626	1.05 (0.42–2.63)	0.908	0.848	1.14 (0.47–2.75)	0.770

Results in bold are statistically significant (*p* < 0.05)

^a^M: major allele, m: minor allele for each SNP

^b^MAF: minor allele frequency

In Table 3 we demonstrate MAF for all nine statistically different SNPs obtained in dbSNP (https://www.ncbi.nlm.nih.gov/SNP/) database for African, European and Asian population. In the same table we demonstrate MAF for both PA patients and controls in the Brazilian population we studied, divided by ethnic ancestry.

**Table 3:** MAF frequency for ethnic ancestry obtained in dbSNP database, PA patients and controls

	MAF frequency								
	dbSNP database			Brazilian PA			Brazilian controls		
SNP	African	European	Asian	African (30)	European (62)	Asian (2)	African (45)	European (206)	Asian (4)
rs3790844	0.13 (6740)	0.23 (158,742)	0.66 (414)	0.23	0.21	1.0	0.26**	0.26	0.88
rs17688601	0.07 (2012)	0.26 (119,368)	0.05 (280)	0.22	0.18	0.00	0.27**	0.25	0.00
rs9854771	0.28 (3086)	0.36 (93,526)	0.14 (238)	0.23	0.27	0.00	0.47*/**	0.38	0.00
rs7310409	0.32 (6064)	0.40 (153,574)	0.38 (370)	0.48	0.47	0.5	0.34	0.42	0.25
rs2941471	0.13 (82)	0.42 (2072)	0.50 (4)	0.65	0.67	0.5	0.41*/**	0.44*	0.375
rs401681	NA	NA	NA	0.42	0.5	0.5	0.45	0.45	0.25
rs13303010	0.64 (3306)	0.10 (117,068)	0.27 (186)	0.35	0.33	0.25	0.35**	0.19*	0.25
rs9543325	0.85 (5580)	0.37 (153,016)	0.46 (370)	0.45	0.49	0.25	0.61*/**	0.40	0.5
rs4795218	0.06 (82)	0.21 (2072)	0.5 (4)	0.13	0.15	0.00	0.14	0.22	0.2

For dbSNP Database values indicated are MAF followed by sample number. For Brazilian PA and controls, MAF are represented according to self-reported ethnic ancestry followed by sample number

^*^Statistical difference (*p* < 0.05) between allele frequency in Brazilian PA patients and controls related to the same ancestry

^**^Statistical difference (*p* < 0.05) between allele frequency in Brazilian controls and dbSNP Database related to the same ancestry

All PRSs were associated with an increase in risk of PA, as expected. When we computed the association between the PRSs and PA risk considering only 67 cases and 228 controls with a call rate of 100%, we observed an OR = 6.83, 95% CI 2.76–16.89, *p* = 3.26 × 10^–5^ for the highest vs. lowest quintile of the unweighted score and OR = 16.77, 95% CI 3.80–74.07, *p* = 1.99 × 10^–4^ for the highest vs. lowest quintile of the weighted score. Results were similar when we considered the whole dataset including 78 cases and 256 controls and “scaled” PRSs (Table 4), as well as when we assembled PRSs with only the 9 SNPs showing association with PA risk in this population (data not shown).

**Table 4:** Associations between PRSs and PA risk

Type of score	Quintiles	OR^a^	95%CI^a^	*p*_value_
Unweighted, subjects with 100% call rate	1	1.00	–	Ref
	2	0.44	0.09–2.25	0.327
	3	2.61	0.97–7.01	0.057
	4	3.29	1.15–9.37	0.026
	5	6.83	2.76–16.89	3.26 × 10^–5^
	Continuous^b^	1.73	1.40–2.15	4.30 × 10^–7^
Unweighted scaled, all subjects	1	1.00	–	Ref
	2	0.65	0.16–2.60	0.539
	3	2.22	0.86–5.73	0.097
	4	4.03	1.57–10.37	0.004
	5	6.70	2.86–15.69	1.21 × 10^–5^
	Continuous^b^	1.72	1.41–2.10	1.02 × 10^–7^
Weighted, subjects with 100% call rate	1	1.00	–	Ref
	2	2.15	0.39–11.75	0.376
	3	3.51	0.71–17.29	0.122
	4	4.90	1.02–23.56	0.047
	5	16.77	3.80–74.07	1.99 × 10^–4^
	Continuous^b^	2.05	1.58–2.65	4.98 × 10^–8^
Weighted scaled, all subjects	1	1.00	–	Ref
	2	1.37	0.41–4.62	0.613
	3	2.07	0.67–6.44	0.209
	4	3.22	1.08–9.60	0.036
	5	8.13	2.95–22.43	5.12 × 10^–5^
	Continuous^b^	1.76	1.42–2.17	2.28 × 10^–7^

M, major allele; m, minor allele for each SNP; MAF, minor allele frequency

^a^OR: odds ratio; CI: confidence interval; all analyses were adjusted for age and sex

^b^The unit for the analysis with the continuous variable was the increment of one quintile

## Discussion

The genetic PA risk factors in SNP context inherent to the Brazilian population have not been studied so far. Here we observed 9 SNPs associated with PA risk (*p* < 0.05) with the most significantly associated being rs3790844, rs9854771, rs2941471, rs401681, rs13303010 and rs9543325. A very important aspect in our results for these SNPs is that for all of them the OR is in the same direction of the original GWAS work.

The first SNP is located at the first intron of *NR5A2*, with MAF in global population of 25%, and we observed a similar value of 27% in our control group, while in PA patients this value was 18%, returning an OR that represents a protective effect of this allele for PA. In a meta-analysis by Chen et al., this SNP had a protective effect in Caucasians, although not in Asian populations [27]. However, another study with 360 pancreatic cancer patients and 400 controls suggested that this SNP is related with pancreatic cancer risk in Japanese subjects [27]. A large study using 3851 pancreatic cancer cases and 3934 controls participants from the previously conducted GWAS in the Pancreatic Cancer Cohort Consortium and the Pancreatic Cancer Case Control Consortium (PanC4) [17, 28] showed this SNP as the most significant risk factor for pancreatic cancer, with an OR of 0.77, again representing a protective effect of minor allele [29].

The SNP rs9854771 has a MAF in global population of 37%. In our control group we observed a similar MAF of 39%, while in PA cases it was 25%. This SNP is located near *TP63* gene, that is a p53 homologue and implicated in tumorigenesis and metastasis [30], and previous GWAS studies have demonstrated significant evidence of association for SNPs in *TP63* in lung cancer and bladder cancer [31–35]. The first description of its role in pancreatic cancer was revealed by Childs[25] with an OR of 0.89 and a subsequent study [36] returned a similar result with an OR 0.76.

A third SNP where the minor allele is associated with a reduction in PA risk is rs2941471 and its MAF in global population is 41%. Here, the control group show a MAF of 44%, while in the PA cases it is 35%. This SNP is located is an intronic region of *HNF4G* gene, at chromosome 8q21.11, which encodes hepatocyte nuclear factor 4 gamma, a transcription factor of the nuclear receptor superfamily whose expression level was increased in five of six clinical human hepatocellular carcinoma samples[37]. When related with pancreas, mice lacking HNF4G have higher numbers of pancreatic β-cells, increased glucose-induced insulin secretion and improved glucose tolerance [38]. A research showing GWAS pathways associated with pancreatic cancer susceptibility factors proposed a link between *HNF4G* inherited variation for pancreatic development [29]. A very consistent research with 2737 pancreatic cancer patients and 4752 controls also yielded this SNP as a genome-wide significant locus (OR = 0.87) [21].

For SNP rs9543325, the global MAF is 38%, similar the frequency of 44% found in our control Brazilian population. In PA, this value increased to 56% and was associated with increased risk for pancreatic cancer in all models analyzed. This association was previously showed in Europeans [28, 39], including Jewish and non-Jewish [40], and in the Taiwanese population [41]. This intergenic SNP maps at 13q22.1 locus, and has been showed to be strongly associated with pancreatic cancer [1, 3, 36, 40–42]. The locus 13q22.1 has other SNPs associated previously with PA, mainly in European and Chinese populations, some studies suggest a potential long-range enhancer activity but mechanisms are still unknown [43].

The SNP rs13303010 has a global population MAF of 12%. In our control group this value was increased to 22% and, among PA patients this frequency increased to 37% and was associated with high cancer risk. The minor allele was also associated with increased PA risk in European [21] and Japanese populations [44]. In European populations, it was highlighted in PA susceptibility only in the largest pancreatic cancer GWAS to date, including 11,537 patients and 17,107 controls from the Pancreatic Cancer Cohort Consortium (PanScan I + II, III), Pancreatic Cancer Case–Control Consortium (PanC4) and PANcreatic Disease ReseArch (PANDoRA) consortium [21]. In the Japanese population, 664 pancreatic cancer cases and 664 controls were analyzed and this SNP was highlighted as PA risk factor [44]. This SNP is mapped at 1p36.33, in the first intron of the *NOC2L* gene and probably influences the host expression. The presence of the risk-increasing allele was associated with higher *NOC2L* expression [21] and this gene encodes the NOC2 like nucleolar associated transcriptional repressor, a protein that represents a novel histone deacetylases-independent inhibitor of histone acetyltransferase [45]. NOC2-like protein has also been associated with the inhibition of p53 and p63 tumor suppressor [46, 47], notably associated with cancer.

The rs401681 is a SNP located in the intron of *CLPTM1L* and 27 kb from the *TERT* gene, being associated with many tumor types [48, 49]. The global population MAF is 43% and, in the Brazilian population, we found a similar frequency of 45% in the control group. In the present study, the presence of the minor allele represents a risk factor for pancreatic cancer. This high risk for PA was also shown in European [17, 39, 50] and Asian populations [51, 52]. It is suggested that rs401681 confers cancer susceptibility by regulating *CLPTM1L* and *TERT* expression [53], both genes implicated in carcinogenesis. *CLPTM1L* gene may be associated in apoptosis processes and high expressed in cisplatin-resistant cell lines [54], *TERT* gene produce catalytic subunit of telomerase, associated with telomere maintaining and usually active in cancer cells [55]. An interesting aspect in rs401681 is that the minor allele is usually associated with increased risk in pancreatic cancer and in melanoma [56] whereas the C allele was associated with increased risk of other tumor types, such as lung, prostate and bladder [48].

Some other SNPs showed statistically significant associations with PA risk in this work. The minor allele of rs17688601, in *SUGCT* gene, and rs4795218, in *HNF1B* gene, were associated with reduced risk in the European population [21, 25]. In the Brazilian population we found them also associated with protection, but only in allelic model. On the other hand, another SNP previously associated SNP in Europeans, the rs7310409 in *HNF1A*, was associated with risk in dominant and co-dominant models, but not in allelic analysis (*p* = 0.065). The other SNPs analyzed were not associated with PA in the Brazilian population in the present study.

Ethnic differences in pancreatic cancer incidence have been reported, especially regarding higher incidence in African in relation with European ancestries [57–59]. Some studies suggested that this higher pancreatic cancer incidence in African ancestry may be partially explained by the greater prevalence of smoking, diabetes, and obesity among these group with no genetic investigation [58, 60]. A recent report demonstrated that family history of pancreatic cancer, diabetes, body mass index ≥ 30 kg/m^2^, current smoking, and red meat intake were associated with pancreatic cancer. More than that, after adjustment for these risk factors, Native Hawaiians, Japanese Americans, and African Americans but not Latino Americans had a higher risk of pancreatic cancer compared to European Americans, showing the genetic influence in pancreatic cancer incidence [61]. Regarding ethnic ancestry on this report, Brazil represents a heterogeneous country with European, African and Asian descendants. Interestingly, statistically significant difference between MAF in PA patients and controls in Brazilian population was observed just in 3 SNPs in African and 2 in European ancestry. In this context, SNPs rs9854771 and rs9543325 were observed as a higher MAF in African ancestry Brazilian controls than PA African Brazilian patients. On the other hand, SNP rs2941471 represent a lower MAF in Brazilian controls than PA patients for both African and European ancestry, the same trend observed for SNP rs13303010 in European ancestry. But when MAF frequency was compared between Brazilian controls and dbSNP data, six SNPs were statistically significantly different, but all in African ancestry. These data demonstrate that self-reported African ancestry from Brazilian controls presents a different genetic SNP profile when compared to African population, probably due to ethnic miscegenation.

PRSs computed with the SNPs we included in the study show a strong association with PA risk when comparing the 20% of the population with highest and lowest PRS values. The small sample size results in very wide confidence intervals of our risk estimates, but the results are in line with a those of a recent study in a much larger population of European origin [62]. It is expected that smaller groups at the extremes of the PRS distribution (e.g. the 5% or 1% with highest/lowest PRS values) will show even more marked differences in risk.

## Conclusion

The main limitation of this study is small sample size. However, as our target SNPs were previously reported as susceptibility loci for PA in large GWAS studies, mainly conducted with European population, this small sample size could establish for the first time SNPs as genetic risk factor for PA in Brazil. Despite a considerable percentage of Amerindian, African and Asian descent in the Brazilian population, the largest ethnic component is European ancestry, showing that genetic risk factors related to Europeans are at least partially reflected in the Brazilian population. This was partially demonstrated by MAF frequency from European ancestry in Brazilian controls and dbSNP database, where no difference was observed. In contrary, Brazilian controls from African ancestry showed MAF statistically significantly different in six out of nine SNPs. Associations of several SNPs reported to affect PA risk in populations of European descent were successfully replicated in our study. Given the limitation of sample size it is not possible to assess whether the SNPs that did not replicate in this work are relevant or not in the Brazilian population. However, it is worth nothing that even for the SNPs that do not reach *p* < 0.05, the direction of the associations (i.e. whether the minor allele is associated with increase or decrease in risk) was consistent with the GWAS data. Our group is recruiting more PA patients and with this data we will have more power in future analyses.

These data can be used for stratification of PA risk, especially in groups that are already known to be at increased risk, such as people with positive family history of pancreatic cancer, and in subjects with high tobacco and alcohol use. PRS can be particularly useful in this context, as shown by our results. More important, this is the first genetic susceptibility study for pancreatic adenocarcinoma in Brazilian population.

## Data Availability

Owing to ethical and legal reasons, providing participants individual raw data of this study are not publicly deposited, and will be made available to researchers who submit a reasonable request to the corresponding author (mateus.aoki@fiocruz.br), conditional to approval by competent IRBs. Data will be stripped from all information allowing identification of study participants. Furthermore, additional work and data analyze is being carried out for further studies.

## Abbreviations

PA: Pancreatic adenocarcinoma
SNP: Single nucleotide polymorphism
GWAS: Genome-wide association studies
PRS: Polygenic Risk Score
OR: Odds ratio
HWE: Hardy–Weinberg equilibrium
MAF: Minor allele frequency
